# Sampling plan in health surveys, city of São Paulo, Brazil, 2015

**DOI:** 10.11606/S1518-8787.2018052000471

**Published:** 2018-09-03

**Authors:** Maria Cecilia Goi Porto Alves, Maria Mercedes Loureiro Escuder, Moises Goldbaum, Marilisa Berti de Azevedo Barros, Regina Mara Fisberg, Chester Luiz Galvão Cesar

**Affiliations:** I Instituto de Saúde. Secretaria de Estado da Saúde de São Paulo. São Paulo, SP, Brasil; II Universidade de São Paulo. Faculdade de Medicina. Departamento de Medicina Preventiva. São Paulo, SP, Brasil; III Universidade Estadual de Campinas. Faculdade de Ciências Médicas. Departamento de Medicina Preventiva e Social. Campinas, SP, Brasil; IV Universidade de São Paulo. Faculdade de Saúde Pública. Departamento de Epidemiologia. São Paulo, SP, Brasil

**Keywords:** Health Surveys, methods, Stratified Sampling, Cluster Sampling, Sample Size, Data Collection, Statistical Analysis, Inquéritos Epidemiológicos, métodos, Amostragem Estratificada, Amostragem por Conglomerados, Tamanho da Amostra, Coleta de Dados, Análise Estatística

## Abstract

**OBJECTIVE:**

To evaluate the sampling plan of the Health Survey of the City of São Paulo (*ISA-Capital 2015*) regarding the accuracy of estimates and the conformation of domains of study by the Health Coordinations of the city of São Paulo, Brazil.

**METHODS:**

We have described the population, domains of study, and sampling procedures, including stratification, calculation of sample size, and random selection of sample units, of the Health Survey of the City of São Paulo, 2015. The estimates of proportions were analyzed in relation to precision using the coefficient of variation and the design effect. We considered suitable the coefficients below 30% at the regional level and 20% at the city level and the estimates of the design effect below 1.5. We considered suitable the strategy of establishing the Health Coordinations as domains after verifying that, within the coordinations, the estimates of proportions for the age and sex groups had the minimum acceptable precision. The estimated parameters were related to the subjects of use of services, morbidity, and self-assessment of health.

**RESULTS:**

A total of 150 census tracts were randomly selected, 30 in each Health Coordination, 5,469 households were randomly selected and visited, and 4,043 interviews were conducted. Of the 115 estimates made for the domains of study, 97.4% presented coefficients of variation below 30%, and 82.6% were below 20%. Of the 24 estimates made for the total of the city, 23 presented coefficient of variation below 20%. More than two-thirds of the estimates of the design effect were below 1.5, which was estimated in the sample size calculation, and the design effect was below 2.0 for 88%.

**CONCLUSIONS:**

The *ISA-Capital 2015* sample generated estimates at the predicted levels of precision at both the city and regional levels. The decision to establish the regional health coordinations of the city of São Paulo as domains of study was adequate.

## RESUMO

**OBJETIVO:**

Avaliar o plano de amostragem do Inquérito de Saúde do Município de São Paulo (ISA-Capital 2015) em relação à precisão das estimativas e à conformação de domínios de estudo pelas coordenadorias de saúde do município de São Paulo.

**MÉTODOS:**

Descrição de população e domínios de estudo, procedimentos de amostragem, incluindo estratificação, cálculo do tamanho da amostra e sorteio de unidades amostrais do Inquérito de Saúde do Município de São Paulo, 2015. As estimativas de proporções foram analisadas em relação à precisão, por meio do coeficiente de variação e do efeito do delineamento. Foram considerados adequados coeficientes menores do que 30% no nível regional e 20% no municipal, e efeitos do delineamento menores do que 1,5. Para considerar adequada a estratégia de estabelecimento das Coordenadorias de Saúde como domínios, foi verificado que, dentro das coordenadorias, as estimativas de proporções para grupos de idade e sexo tinham a precisão mínima aceitável. Os parâmetros estimados referiram-se aos temas: uso de serviços, morbidade e autoavaliação em saúde.

**RESULTADOS:**

Foram sorteados 150 setores censitários, 30 em cada Coordenadoria de Saúde, sorteados e visitados 5.469 domicílios ocupados, e realizadas 4.043 entrevistas. Das 115 estimativas feitas para os domínios de estudo, 97,4% apresentaram coeficientes de variação menores do que 30% e 82,6% menores do que 20%. Das 24 estimativas feitas para o total do município, 23 apresentaram coeficiente de variação menor do que 20%. Mais de dois terços das estimativas do efeito do delineamento foram inferiores a 1,5, valor previsto no cálculo do tamanho da amostra, e o efeito do delineamento foi menor do que dois para 88%.

**CONCLUSÕES:**

A amostra do ISA-Capital 2015 gerou estimativas situadas nos patamares previstos de precisão, tanto as de nível municipal como regional. Foi acertada a decisão de estabelecer as coordenadorias regionais de saúde do município de São Paulo como domínios de estudo.

## INTRODUCTION

It is important to know the sampling plans used in epidemiological surveys and the evaluation of the alternatives applied to improve the practice of household surveys. There are few publications on this subject in the Brazilian literature to support new experiences^1^ ^-^ ^6^. It is particularly interesting the provision of subsidies to improve sampling designs in time trend studies, which are based on data from successive surveys. More such studies have been carried out in recent years^7^ ^-^ ^10^.

In cities in the State of São Paulo, Brazil, health surveys called ISA have been carried out since 2001. The objective is to evaluate the health status of the population living in the city, according to their living conditions, addressing aspects related to lifestyle, acute and chronic morbidities, preventive practices, and use of health services^11^. They are conducted by a team of researchers from public universities of São Paulo and the State Department of Health of São Paulo. Editions were carried out in 2003^a^, 2008^b^, and 2015^c^, in the city of São Paulo with most support from the City Health Department, and in 2001, 2008^d^, and 2014/2015 in the city of Campinas^e^.

In these surveys, probabilistic sampling is used, always seeking inferences to the study population based on measures of precision. Although they are similar, the sampling plans used in the different years of the ISA-Capital have different aspects. Their adoption was motivated by the desire to improve the process of data collection based on acquired experiences, preserving the possibility of comparison between the different editions.

The planning of the 2015 survey was based on the interest to produce information on smaller areas of the city, which are more homogeneous in relation to the epidemiological profile. Consistent with this objective, the City Health Department, the main funder of the project, intended to reinforce the use of results by regional managers. This confluence of interests culminated in the definition of regional Health Coordinations of the city of São Paulo^f^ as domains of study in the ISA-Capital 2015.

The objective of this study was to evaluate the sampling plan of the ISA-Capital 2015 regarding the precision of estimates and the conformation of the domains of study by the Health Coordinations of the city of São Paulo, Brazil.

## METHODS

Below, we describe the sampling plan of ISA-Capital 2015, highlighting the following aspectcs: population and domains of study and sampling procedures, including calculation of sample size, and random selection of sample units. In addition, we present the results of the application of the sampling plan, considering the households visited and the interviews obtained.

The estimates obtained with the ISA-Capital 2015 sample for the parameters of interest were analyzed for precision using the coefficient of variation. Estimates with coefficients below 20% for the city level and below 30% for the regional were considered sufficiently precise. Thus, we would consider as suitable the establishment of the Health Coordinations as the domains of study if the estimates of proportions according to the age and sex domains had minimum acceptable precision within the coordinations, indicated by coefficients of variation below 30%.

We also evaluated the measures of effect of design, widely used as measures of efficiency of complex sampling designs^12^ ^,^ ^13^. Those below 1.5 were considered suitable, which was adopted in the planning of the sample. We also verified the frequency of estimates below 2.0, which is frequently adopted in sampling plans^3^ ^,^ ^4^ ^,^ ^6^ ^,^ ^14^.

The parameters estimated in this study were the prevalence of persons who reported the following: use of health service in the last 30 days, hospitalization in the last year, visit to the dentist in the last year, hypertension, allergy, health problem in the last 15 days, and excellent or good self-assessment of health. These parameters were related to the following subjects: use of services, morbidity, and self-assessment of health, usually studied in health surveys. The reference to allergy was selected because it was the only morbidity in which the estimates for adolescents were greater than 10% for the most part.

The reference population of the ISA-Capital 2015 consisted of individuals aged 12 years or more living in permanent private households in the urban area of the city of São Paulo (Table 1, block 1)^g^. For the delimitation of the population, the survey used the census tracts classified in the 2010 Census as urban situation – urbanized area, non-urbanized area, and isolated urbanized area – and ‘common’ and ‘special subnormal’ types.

**Table 1:** Reference population, planned sample of persons and households, and person/household ratio according to age and sex groups and Health Coordination. São Paulo, State of São Paulo, Brazil, ISA-Capital 2015.

Coordination	Age (years)/Sex				Total
	12 to 19	20 to 59		60 or more	
		Men	Women		
Block 1 – Population in 2010 (aggregate of urban census tracts)					
North	268,489	613,562	684,973	266,052	1,833,076
Central-West	155,753	464,696	518,726	238,299	1,377,474
Southeast	277,068	755,332	848,308	403,229	2,283,937
South	335,585	706,625	784,700	217,103	2,044,013
East	304,317	617,650	682,615	206,808	1,811,390
Total	1,341,212	3,157,865	3,519,322	1,331,491	9,349,890
Block 2 – Sample that would be obtained with proportional sharing by age/sex domain					
North	124	285	318	123	850
Central-West	96	287	320	147	850
Southeast	103	281	316	150	850
South	140	294	326	90	850
East	143	290	320	97	850
Total	606	1,436	1,600	608	4,250
Block 3 – Planned sample					
North	153	234	261	203	850
Central-West	150	223	249	228	850
Southeast	150	219	246	234	850
South	176	247	275	152	850
East	179	242	267	162	850
Total	808	1,165	1,298	980	4,250
Block 4 – Person/household ratio					
North	0.3994	0.9128	1.0191	0.3958	-
Central-West	0.2724	0.8126	0.9071	0.4167	-
Southeast	0.3151	0.8591	0.9649	0.4586	-
South	0.4410	0.9286	1.0312	0.2853	-
East	0.4598	0.9332	1.0313	0.3125	-
Total	-	-	-	-	-
Block 5 – Households required for the planned interviews					
North	383	256	256	513	513
Central-West	551	274	275	547	551
Southeast	476	255	255	510	510
South	399	266	267	533	533
East	389	259	259	518	518
Total	2,198	1,311	1,311	2,621	2,625
Block 6 – Households to be randomly selected according to non-response					
North	709	475	474	950	950
Central-West	1,020	508	508	1,013	1,020
Southeast	881	472	472	945	944
South	739	493	494	987	987
East	721	480	479	960	959
Total	4,071	2,428	2,428	4,855	4,861
Block 7 – Households required by census tracts					
North	23.6	15.8	15.8	31.7	31.7
Central-West	34.0	16.9	16.9	33.8	34.0
Southeast	29.4	15.7	15.7	31.5	31.5
South	24.6	16.4	16.5	32.9	32.9
East	24.0	16.0	16.0	32.0	32.0
Total	27.1	16.2	16.2	32.4	32.4
Block 8 – Inverse of the planned sampling fractions					
North	947.61	1415.91	1417.19	707.72	
Central-West	560.71	1125.27	1124.95	564.39	
Southeast	997.44	1862.46	1862.14	930.53	
South	1029.64	1544.85	1540.87	771.29	
East	918.05	1378.23	1380.57	689.36	

ISA: Health Survey of the City of São Paulo

Stratified sampling was used and clusters were selected in two stages: census tracts and households.

The strata were formed by the five Health Coordinations of the city of São Paulo: North, Central-West, Southeast, South, and East, which were domains of study. For the sample planning, we also considered the age and sex groups as domains: adolescents (12 to 19 years), male adults (men aged 20 to 59 years), female adults (women aged 20 to 59 years), and older adults (60 years or more). We defined 20 domains of study, both geographic and demographic.

For operational reasons, the total sample size would be 4,250 persons. In order for the Health Coordinations to have the same potential for data analysis, 850 persons were assigned to each one. The sample would have the distribution presented in Table 1 (block 2) if the distribution by the age and sex domains were proportional to the population of these domains in each Coordination. However, the participation of the “adolescent” and “older adult” groups was changed in the sample for more precise estimates in these domains. A 50% larger adolescent population was considered, as well as a 100% larger older population, and a new distribution of the sample was carried out. The numbers of interviews was increased to 150 for two domains: adolescents of the Central-West and Southeast Coordinations (Table 1, block 3).

This number could allow the estimation of proportions of 0.50, with a sampling error of 0.10, considering a 95% confidence level and a design effect of 1.5. The calculation was carried out from the algebraic expression that determines the minimum sample size to estimate proportions under complex samples^13^ ^,^ ^15^: $n=\frac{P\times (1-P)}{{\left(d/z\right)}^{2}}\times deff$, where *n* is the sample size, *P* is the parameter to be estimated, *z* = 1.96 is the value in the reduced normal curve related to the 95% confidence level of the confidence intervals, *d* is the sampling error, and *deff* is the effect of the design.

The expected mean number of persons per household (ratio between persons and households) was calculated in each domain from the 2010 Census data (Table 1, block 4) to determine the number of households that interviews should be conducted. The number of households was obtained by dividing the sample size of each domain by the respective ratio between persons and households (Table 1, block 5).

However, in order to reached the minimum number of interviews in the presence of non-response (vacant or closed households, refusals, or households with a resident unable to respond), the inclusion of a larger number of households in the sample was planned (Table 1, block 6). A non-response rate of 40% and a percentage of vacant households of 10% were considered.

The interviewees were randomly selected using two-stage sampling. In the first stage, 30 census tracts were randomly selected in each Coordination, with probability proportional to size, measured by the number of permanent private households counted in the 2010 Census, sorted by the average per capita income of the households in the tract.

In the second stage, the households were selected using two different random selections. In tracts classified by the IBGE^g^ as “common”, the households were systematically selected, based on the list of households carried out in the field. In the census tracts classified as “special subnormal” (which corresponds to *favela* slums in the city of São Paulo), segments of households were created (mean size of six households). These segments were the second stage of selection, in which the random selection of six segments per tract was planned.

The households were randomly selected corresponding to the rarest domain (adolescents in the Central-West region and older adults in the other four Coordinations) in each tract, and this sample was called the main sample. From the main sample, sub-samples were randomly selected with sizes defined for the other age and sex domains (Table 1, block 7). This type of random selection is equivalent to obtaining four concomitant samples, related to the four domains of study.

There was no intra-household random selection. All persons belonging to the domain for which the household was selected were included in the sample. In the data collection equipment of the interviewers, it was indicated the domains to be searched in each household of the sample.

The overall sampling fractions in each Coordination were:

– in the rarest domain: $f=\frac{30\times M_{i}}{M}\times \frac{b}{M_{i}}$
– in the other domains: $f=\frac{30\times M_{i}}{M}\times \frac{b}{M_{i}}\times \frac{b_{domínio}}{b}$

where *M*_i_ is the number of households in tract *i* (data from the 2010 Census), *M* is the total number of households in the Coordination (data from the 2010 Census), *b* is the number of households in the rarest domain, i.e., the main sample, and *b*_domain_ is the number of households required for each of the three less rare domains.

The second-stage sampling fraction was fixed, which increased (or decreased) the number of households randomly selected in relation to what was planned if the census tract had grown (or decreased) since the 2010 Census. With this option, the second-stage sampling fraction can be rewritten as: $\frac{b({M_{i}}^{'}/M_{l})}{{M_{i}}^{'}}$, where *M*_i_^'^ is the number of households in tract *i* obtained in the listing of households, performed in the field.

In order to compensate for the differences between the probabilities of the random selection of the individuals in the sample, design weights were introduced in the data analysis step, expressed as the inverse of the sampling fractions, *F=1/f* (Table 1, block 8)^16^. This weight can be interpreted as the number of persons in the population “represented” for each person randomly selected.

## RESULTS

The fieldwork of ISA-Capital began in the second half of 2014, but 80.0% of the interviews were conducted in 2015, between January and December. A total of 5,942 households were effectively selected and visited. Of these, 8.0% were vacant households, which amounted to 5,469 occupied houses (Table 2). Information could be obtained in 76.4% of the occupied households about the residents and the presence of persons belonging to the age and sex groups of interest. In these households, 73.4% of the eligible residents were interviewed.

**Table 2:** Occupied households visited, interviews conducted, and mean interviews by census tract, according to age and sex groups and Health Coordination. São Paulo, State of São Paulo, Brazil, ISA-Capital 2015.

Coordination		Age (years)/sex				Total
		12 to 19	20 to 59		60 or more	
			Men	Women		
Occupied households (among those randomly selected and visited)						
	North	660	449	437	791	1,004
	Central-West	989	500	502	924	1,186
	Southeast	949	506	495	889	1,192
	South	760	498	513	900	1,124
	East	647	426	402	732	963
	Total	4,005	2,379	2,349	4,236	5,469
Interviews conducted						
	North	161	200	229	187	777
	Central-West	113	135	160	201	609
	Southeast	150	182	231	260	823
	South	244	231	301	199	975
	East	191	205	291	172	859
	Total	859	953	1,212	1,019	4,043
Interviews conducted by tract						
	North	5.4	6.7	7.6	6.2	25.9
	Central-West	3.8	4.5	5.3	6.7	20.3
	Southeast	5.0	6.1	7.7	2.0	27.4
	South	8.1	7.7	10.0	6.6	32.5
	East	6.4	6.8	9.7	5.7	28.6
	Total	5.7	6.4	8.1	6.8	27.0

ISA: Health Survey of the City of São Paulo

The number of households randomly selected was higher than that considered necessary for the interviews (n = 4,831), provided for in the sampling plan. Nevertheless, the number of interviews was smaller than planned. The minimum of 150 interviews was not reached in two domains (adolescents and adult males in the Central-West Coordination). The Coordinations that had a smaller number of interviews were North (9.0% smaller) and Central-West (28.0% smaller). The target number of interviews for the total of the city was reached for adolescents and older adults and it was further from what was proposed for the group of adult males (18.0% smaller).

Of the 115 estimates made for the domains of study, 97.4% presented coefficients of variation below 30.0%, and 82.6% were below 20.0% (Tables 3 and 4). Of the few estimates that did not reach the desired level of precision (three estimates), one was obtained with a small sample of interviews, below 150, and one estimate was below 0.10, which means an event of very small frequency. All prevalence estimates above 0.30 showed low coefficients of variation; the inverse happened for the estimates below 0.10; none reached the desired levels of accuracy. Of the 24 estimates made for the city, almost all (23 estimates) showed a coefficient of variation below 20%.

**Table 3:** Number of interviews, prevalence estimates, confidence intervals, coefficients of variation, and effects of design, among men and women aged 20 to 59 years. São Paulo, State of São Paulo, Brazil, ISA-Capital 2015.

Indicators	Domain	Men					Women				
	Region	n	p	95%CI	cv	*deff*	n	p	95%CI	cv	*deff*
Use of a health service in the last 30 days	North	198	20.6	14.2–26.9	15.6	1.246	228	36.3	30.0–42.7	8.9	1.016
	Central-West	135	24.4	17.0–31.8	15.4	1.026	158	35.7	27.0–44.4	12.3	1.319
	Southeast	181	29.9	20.5–39.2	15.8	1.920	231	36.4	29.8–43.0	9.2	1.106
	South	229	18.6	13.6–23.7	13.8	0.990	301	35.6	27.1–44.0	12.0	2.391
	East	205	29.3	21.4–37.3	13.7	1.595	291	39.9	33.3–46.4	8.3	1.320
	Total	948	24.6	21.2–28.0	7.0	2.171	1,209	36.8	33.5–40.1	4.5	1.770
Visit to the dentist in the previous year	North	198	59.6	50.0–69.2	8.2	1.927	228	71.4	66.1–76.6	3.7	0.775
	Central-West	134	70.4	62.3–78.5	5.8	1.062	159	77.0	68.4–85.7	5.7	1.705
	Southeast	182	65.6	57.5–73.8	6.3	1.366	230	70.6	61.9–79.2	6.2	2.114
	South	229	52.2	45.4–59.1	6.6	1.096	300	60.2	53.6–66.8	5.5	1.390
	East	203	58.5	51.3–65.7	6.2	1.094	290	59.8	52.8–66.7	5.9	1.480
	Total	946	60.5	56.8–64.2	3.1	1.373	1,207	66.9	63.6–70.3	2.5	1.551
Excellent or good self-assessment of health	North	199	80.6	74.1–87.1	4.1	1.385	229	64.9	55.8–74.0	7.1	2.118
	Central-West	134	84.7	78.4–91.0	3.8	1.050	160	77.5	71.6–83.4	3.9	0.824
	Southeast	182	81.6	73.7–89.6	4.9	1.965	231	73.5	66.2–80.8	5.0	1.599
	South	229	77.1	71.8–82.3	3.4	0.913	300	65.8	59.2–72.5	5.1	1.505
	East	205	77.4	72.3–82.6	3.3	0.779	291	66.6	61.2–71.9	4.1	0.959
	Total	949	79.9	77.0–82.8	1.8	1.815	1,211	69.1	65.9–72.4	2.4	1.884
Health problem in the last 15 days	North	199	16.4	12.3–20.5	12.7	0.628	229	24.7	19.1–30.2	11.4	0.976
	Midwest	135	15.9	11.2–20.6	15.0	0.567	160	20.1	14.4–25.8	14.4	0.827
	Southeast	182	13.4	8.5–18.2	18.2	0.929	231	26.6	19.8–33.4	12.9	1.395
	South	231	9.5	5.2–13.9	22.9	1.271	301	13.9	8.4–19.4	20.1	1.964
	East	205	21.8	16.5–27.0	12.2	0.848	291	21.0	15.8–26.2	12.5	1.195
	Total	952	15.2	13.0–17.3	7.3	1.281	1,212	21.2	18.6–23.9	6.3	1.615
Hypertension	North	200	13.6	7.0–20.1	24.5	1.882	228	23.3	17.1–29.5	13.4	1.239
	Central-West	134	10.6	4.1–17.1	31.0	1.525	159	11.1	3.8–18.5	33.5	2.233
	Southeast	182	13.8	9.3–18.2	16.4	0.780	231	15.5	10.3–20.6	16.9	1.205
	South	231	14.0	9.9–18.2	15.1	0.861	301	20.5	15.5–25.4	12.2	1.154
	East	204	15.7	10.9–20.6	15.7	0.928	291	18.0	13.1–22.9	13.8	1.206
	Total	951	13.8	11.4–16.1	8.6	1.133	1,210	18.1	15.6–20.6	7.0	1.301
Allergy	North	199	16.8	9.5–24.2	22.1	1.969	228	19.9	13.7–26.1	15.8	1.402
	Central-West	134	15.2	8.2–22.0	23.1	1.275	160	18.5	11.2–25.8	20.0	1.448
	Southeast	181	13.3	8.4–18.4	18.6	0.961	229	17.7	13.1–22.3	13.1	0.842
	South	230	5.3	1.3–8.8	32.3	1.353	300	10.1	5.2–15.0	24.5	2.011
	East	205	14.0	9.9–18.2	14.8	0.732	291	15.6	12.0–19.2	11.8	0.741
	Total	949	12.6	10.1–15.0	9.7	1.281	1,208	16.0	13.7–18.4	7.4	1.246

ISA: Health Survey of the City of São Paulo; cv: coefficient of variation; *deff*: effect of the design

**Table 4:** Number of interviews, prevalence estimates, confidence intervals, coefficients of variation, and effects of design in adolescents aged 12 to 19 years and older adults aged 60 years or more. São Paulo, State of São Paulo, Brazil, ISA-Capital 2015.

Indicators	Domain	Adolescents					Older adults				
	Region	n	p	95%CI	cv	*deff*	n	p	95%CI	cv	*deff*
Use of a health service in the last 30 days	North	158	24.2	18.2–30.2	12.5	0.782	187	42.5	37.2–47.7	6.3	0.541
	Central-West	112	21.4	11.3–31.5	23.8	1.710	199	39.5	32.6–46.4	8.8	0.999
	Southeast	149	24.5	17.6–31.5	14.4	0.992	260	41.4	32.5–50.3	10.9	2.179
	South	242	19.6	15.1–24.1	11.7	0.804	199	43.7	36.0–51.3	8.8	1.200
	East	189	22.1	16.1–28.1	13.7	1.005	170	37.1	29.5–44.7	10.4	1.078
	Total	850	22.3	19.5–25.1	6.4	0.628	1,015	41.0	37.4–44.5	4.4	0.855
Visit to the dentist in the previous year	North	158	65.5	56.5–74.4	6.9	1.421	178	42.3	33.3–51.4	10.8	1.512
	Central-West	113	79.3	68.9–89.6	6.6	1.863	197	61.1	50.5–71.7	8.8	2.387
	Southeast	150	59.7	50.5–68.9	7.8	1.341	258	47.7	36.3–59.0	12.0	3.380
	South	243	60.5	52.7–68.3	6.5	1.580	196	41.8	32.1–51.5	11.7	1.921
	East	188	57.6	51.1–64.2	5.7	0.832	166	37.7	29.3–46.1	11.3	1.264
	Total	852	62.3	58.5–66.2	3.1	1.345	995	46.4	41.5–51.2	5.3	1.535
Self-assessment of excellent or good health	North	161	87.5	83.5–91.5	2.3	0.608	186	60.2	49.7–70.8	8.9	2.225
	Central-West	113	82.4	75.0–89.8	4.5	1.092	201	74.2	67.0–81.3	4.9	1.367
	Southeast	150	80.3	73.6–86.9	4.2	1.082	260	67.2	60.9–73.4	4.7	1.176
	South	243	84.8	80.2–89.3	2.7	0.999	199	56.8	49.0–64.6	7.0	1.259
	East	191	81.3	74.0–88.6	4.6	1.712	172	52.0	43.5–60.5	8.2	1.256
	Total	858	83.3	80.6–86.1	1.7	0.777	1,018	62.7	59.0–66.5	3.0	0.997
Health problem in the last 15 days	North	161	16.6	10.7–22.4	17.8	1.010	187	24.9	16.9–32.9	16.2	1.622
	Central-West	113	13.2	6.3–20.2	26.4	1.194	201	13.9	8.0–19.8	21.6	1.504
	Southeast	150	19.7	11.4–28.0	21.3	1.656	258	20.4	15.3–25.5	12.7	1.057
	South	243	10.4	4.9–15.9	26.6	2.000	199	26.6	13.7–39.5	24.5	4.303
	East	191	19.5	13.7–25.4	15.1	1.049	172	30.2	22.8–37.7	12.5	1.159
	Total	858	16.03	13.0–19.0	9.42	0.922	1,017	22.9	19.3–26.5	8.0	1.216
Hypertension	North	161	…	…	…	…	187	49.2	41.2–57.2	8.3	1.232
	Central-West	113	…	…	…	…	201	56.7	44.4–69.0	11.0	3.168
	Southeast	149	…	…	…	…	259	62.3	55.8–68.7	5.2	1.159
	South	242	…	…	…	…	199	46.8	38.2–55.4	9.3	1.509
	East	191	…	…	…	…	171	55.2	50.1–60.4	4.7	0.465
	Total	856	1.14	0.3–2.0	37.33	1.371	1,017	54.8	51.0–58.7	3.5	1.525
Allergy	North	161	25.6	19.3–31.9	12.5	0.869	187	14.0	8.9–19.1	18.4	1.028
	Central-West	113	24.4	12.1–36.6	25.5	2.373	201	23.5	14.6–32.4	19.2	2.258
	Southeast	149	18.5	9.7–27.3	24.0	1.958	260	17.3	12.5–22.1	14.0	1.065
	South	244	8.9	4.2–13.6	26.7	1.704	198	8.9	4.2–13.7	27.0	1.407
	East	191	25.9	20.0–31.9	11.6	0.901	171	20.4	13.8–27.0	16.3	1.154
	Total	858	19.8	16.6–23.0	8.3	1.439	1,017	16.8	14.0–19.6	8.4	1.455

ISA: Health Survey of the City of São Paulo; cv: coefficient of variation; *deff*: effect of the design

More than two-thirds (69.0%) of the estimates of the design effect were below 1.5, which was estimated in the sample size calculation, and the design effect was below 2.0 for 88.0%.

The mean number of interviews per tract for the age and sex groups for the set of Coordinations ranged from 5.7 to 8.1.

## DISCUSSION

The ISA-Capital 2015 sample generated estimates at the predicted levels of precision at both the city and regional levels, which indicates that the decision to establish the regional health coordinations of the city of São Paulo as domains of study was adequate.

There is no single criterion adopted universally to establish a limit for the values of coefficient of variation. Several factors must be considered. The knowledge on whether a particular coefficient of variation is too high or too low requires experience on similar data^17^. The Fundação Sistema Estadual de Análise de Dados (SEAD), responsible for several surveys in the State of São Paulo, guides its decision according to the frequency of the survey and the nature of the phenomenon under study. It does not adopt a single policy for the dissemination of the results of the research it carries out^h^. Thus, different limits for the coefficient of variation were stipulated in the various surveys conducted^i^. When disclosing the results of the Household Expenditure Survey of 2015/2016, the National Statistical Institute of Portugal proposed that estimates with coefficients of variation between 20% and 30% should be carefully used and those with coefficients above 30% should be disregarded^j^. These limits, as well as those proposed in other health studies^3^ ^,^ ^6^, coincide with those adopted in our study.

The number of households effectively selected was higher than planned. The use of constant fractions in the random selection in the second sampling step may be responsible for this result. With this strategy, the 38% increase in the number of households between the Census and the survey data collection was reflected in the number of households sampled. The equiprobability of the sample was kept by the random selection with probability proportional to size, sacrificing control over its final size.

In addition, sampling fractions were changed in the tracts not yet visited when the follow-up of the field work detected that the non-response rates were greater than expected. This further increased the number of households randomly selected. These increases were offset by the use of weights in the data analysis.

The follow-up of the field work by the team responsible for the survey was carried out through spreadsheets, whose models were improved throughout the various editions of the ISA project. The detailing of the response rates at the household and resident levels by census tract together with the interviews allowed problems to be detected as soon as they occurred. This helped the introduction of adjustments in the sampling plan.

The number of interviews was lower than planned, which shows that population participation in the survey was lower than expected. All households were visited at least three times, at different times and days, which did not prevent high non-response rates.

Although the sample size of the ISA-Capital 2015 is similar to previous editions, the field work was extended for a longer period, mainly due to the greater number of census tracts selected (150 in 2015, 80 in 2008, and 60 in 2003). This was a necessity created by the option of adopting the Health Coordinations as domains of study, setting the number of tracts to 30 in each one. It can be understood as the cost of obtaining regional estimates in this edition of the ISA.

The increase in the number of census tracts meant a smaller number of interviews by tract: 5.7 to 8.1, on average, by age and sex domain. These numbers are far from the optimal number of interviews in each primary sampling unit. This number seeks the balance between precision and cost, considering the ratio between the costs of including a new conglomerate and a new household in the sample, in addition to the degree of intra-cluster homogeneity^18^ ^,^ ^k^. For a 20-fold cost of including a new conglomerate compared to including a new interview^19^, and considering a degree of homogeneity of 0.05^l^, the indicated number of interviews for each tract would be 20. However, the decrease in the concentration of interviews by tract, although increasing the cost, had the advantage of increasing precision. This contributed to the small effect of design. The random selection of cluster as opposed to simple selection often increases the variance of the estimates according to the intraclass correlation, which is a characteristic of the population that cannot be changed by the sampling process. However, the inclusion of fewer elements per cluster in the sample can reduce the impact of intraclass correlation on variance, leading to smaller estimates for the design effect.

The random selection adopted in the ISA-Capital, in which the four samples related to the four age and sex domains are obtained simultaneously, relativizes the importance of the increase in cost. The number of interviews per tract, considering the four domains, was between 20.3 and 32.5, depending on the Coordination.

One of the consequences of using weights in the data analysis step is the increase of the estimates of the design effect, and the increase is proportional to the variation between applied weights^20^. In the first ISA editions, sample size was the same for all age and sex domains. This resulted in very different weights, which impacted the design effect when more than one domain was analyzed together. The 2015 survey sought the closer proportional distribution of the sample by the age and sex domains in each Coordination, avoiding the previously observed discrepancy between weights.

One of the characteristics common to all issues of the ISA-Capital is the non-use of intra-household selection. In terms of efficiency, the strategy of interviewing all residents belonging to the age and sex group of interest is superior to that in which only one of the residents of the household is randomly selected for the interview^21^. To apply it, the option of the ISA is to select randomly a main sample and, from it, obtain subsamples of households, according to the need of each domain defined based on the mean number of persons per household indicated in the Census. With the adequate number of households for each domain, there is no need for an intra-household selection.

Based on data from previous editions of the ISA, Alves et al.^22^ have shown that it is particularly advantageous to use segments as an alternative to full address listing when applied to the random selection of households in favela slums. In the ISA-Capital 2015, in addition to being used in favela slums, this strategy was also applied in the last tracts to fasten the field work. Among the advantages associated with the use of segments, we can highlight the speed in locating and identifying households.

The estimates of prevalence within Health Coordinations, for the most part, were considered suitable for all age and sex groups defined as a domain in the ISA-Capital 2015. This result allows the use of data from the survey by health managers in the city of São Paulo, who will have regional information that is sufficiently precise to assess issues related to reported morbidity and the use of services. However, it is important to be aware of the use of results related to rare events, especially when done with small samples.

It was a good choice to follow this path so that the sample in this edition of ISA-Capital could have the data disaggregated by Health Coordinations. The comparison of the results of different regions of the city can help in the understanding of the determinants of the epidemiological situation of the resident population and aspects related to the use of health services available in the area. The government of the city of São Paulo has prepared reports that analyze the data related to the various subjects addressed in the research^m^. This production shows the potential contribution of the survey in the analysis of health problems of the population of the city and the adequacy of the coping strategies adopted. The repetition of surveys in the city meets the interest of studying trends in several measures related to the health of the population living in it.

## Footnotes

a Cesar CLG, Carandina L, Alves MCGP, Barros MBAB, Goldbaum M. Inquéritos de Saúde no Município de São Paulo - ISA-Capital 2003. São Paulo: USP/FSP; 2003 [cited 2017 Apr 4]. Available from: http://www.fsp.usp.br/isa-sp/old/index_arquivos/Page3157.htm

b Cesar CLG, Carandina L, Alves MCGP, Barros MBAB, Goldbaum M. Inquéritos de Saúde no Município de São Paulo - ISA-Capital 2008. São Paulo: USP/FSP; 2008 [cited 2017 Apr 4]. Available from: http://www.fsp.usp.br/isa-sp/old/index_arquivos/Page1494.htm

c Seminário Inquérito de Saúde: ISA-Capital 2015; 31 mar 2016; São Paulo, SP. São Paulo: FSP-USP; c2010 [cited 2017 Apr 4]. Available from: http://www.fsp.usp.br/site/eventos/mostrar/5523

d UNICAMP, Faculdade de Ciências Médicas, Centro Colaborador em Análise da Situação de Saúde. Inquérito de Saúde do Município de Campinas – ISACamp 2008. Campinas: CCAS; 2008 [cited 2017 Apr 4]. Available from: http://www.fcm.unicamp.br/fcm/ccas-centro-colaborador-em-analise-de-situcao-de-saude/isacamp/2008

e UNICAMP, Faculdade de Ciências Médicas, Centro Colaborador em Análise da Situação de Saúde. Inquérito de Saúde do Município de Campinas – ISACamp 2014/2015. Campinas: CCAS; 2014 [cited 2017 Apr 4]. Available from: http://www.fcm.unicamp.br/fcm/ccas-centro-colaborador-em-analise-de-situcao-de-saude/isacamp/2014

f Prefeitura de São Paulo. Secretaria Municipal de Saúde: organização. São Paulo; c2017 [cited 2018 Feb 1]. Available from: http://www.prefeitura.sp.gov.br/cidade/secretarias/saude/organizacao/

g Instituto Brasileiro de Geografia e Estatística. Censo Demográfico 2000: agregado por setores censitários dos resultados do universo. 2.ed. Rio de Janeiro: IBGE; 2003 [cited 2017 Apr 4]. Available from: ftp://ftp.ibge.gov.br/Censos/Censo_Demografico_2000/Dados_do_Universo/Agregado_por_Setores_Censitarios

h Dini NP. Pesquisa por amostragem: política de divulgação de estimativas com baixa precisão amostral. Rio de Janeiro: IBGE; s.d. [cited 2017 Apr 4]. Available from: https://www.ibge.gov.br/confest_e_confege/pesquisa_trabalhos/CD/mesas_redondas/294-1.pdf

i Instituto Brasileiro de Geografia e Estatística. Pesquisa de Condições de Vida - PCV 2006. Rio de Janeiro: IBGE; 2006 [cited 2017 Apr 22]. Available from: http://produtos.seade.gov.br/produtos/pcv/pdfs/aspectos_metodologicos_pcv2006.pdf

j Instituto Nacional de Estatística (PT). Orçamentos Familiares: inquérito às despesas das famílias – 2015-2016. Lisboa: INE; 2017 [cited 2017 Apr 22]. Available from: https://www.ine.pt/xportal/xmain?xpid=INE&xpgid=ine_publicacoes&PUBLICACOESpub_boui=277098526&PUBLICACOESmodo=2&xlang=pt

k Where *C*_1_ is the cost of an additional tract and *C*_2_ is an additional interview, and ρ is the degree of intra-cluster homogeneity.

j Value based on results observed in a previous survey carried out in the city of São Paulo (ISA-Capital), in which most of the health variables studied presented values below 0.05.

m Prefeitura de São Paulo, Secretaria Municipal de Saúde. Publicações sobre ISA-Capital - SP. São Paulo; 2014 [cited 2017 Apr 4]. Available from: http://www.prefeitura.sp.gov.br/cidade/secretarias/saude/epidemiologia_e_informacao/isacapitalsp/index.php?p=177260
